# Early weight-bearing after periacetabular osteotomy leads to a high incidence of postoperative pelvic fractures

**DOI:** 10.1186/1471-2474-15-234

**Published:** 2014-07-11

**Authors:** Hiroshi Ito, Hiromasa Tanino, Tatsuya Sato, Yasuhiro Nishida, Takeo Matsuno

**Affiliations:** 1 Department of Orthopaedic Surgery, Asahikawa Medical University, Higashi 2-1-1-1, Midorigaoka, 078-8510 Asahikawa, Japan

**Keywords:** Periacetabular osteotomy, Accelerated rehabilitation protocol, Complications

## Abstract

**Background:**

It has not been shown whether accelerated rehabilitation following periacetabular osteotomy (PAO) is effective for early recovery. The purpose of this retrospective study was to compare complication rates in patients with standard and accelerated rehabilitation protocols who underwent PAO.

**Methods:**

Between January 2002 and August 2011, patients with a lateral center-edge (CE) angle of < 20°, showing good joint congruency with the hip in abduction, pre- or early stage of osteoarthritis, and age younger than 60 years were included in this study. We evaluated 156 hips in 138 patients, with a mean age at the time of surgery of 30 years. Full weight-bearing with two crutches started 2 months postoperatively in 73 patients (80 hips) with the standard rehabilitation protocol. In 65 patients (76 hips) with the accelerated rehabilitation protocol, postoperative strengthening of the hip, thigh and core musculature was begun on the day of surgery as tolerated. The exercise program included active hip range of motion, and gentle isometric hamstring and quadriceps muscle sets; these exercises were performed for 30 minutes in the morning and 30 minutes in the afternoon with a physical therapist every weekday for 6 weeks. Full weight-bearing with two axillary crutches started on the day of surgery as tolerated. Complications were evaluated for 2 years.

**Results:**

The clinical results at the time of follow-up were similar in the two groups. The average periods between the osteotomy and full-weight-bearing walking without support were 4.2 months and 6.9 months in patients with the accelerated and standard rehabilitation protocols (P < 0.001), indicating that the accelerated rehabilitation protocol could achieve earlier recovery of patients. However, postoperative fractures of the ischial ramus and posterior column of the pelvis were more frequently found in patients with the accelerated rehabilitation protocol (8/76) than in those with the standard rehabilitation protocol (1/80) (P = 0.013).

**Conclusion:**

The accelerated rehabilitation protocol seems to have advantages for early muscle recovery in patients undergoing PAO; however, postoperative pelvic fracture rates were unacceptably high in patients with this protocol.

## Background

The efficacy of an accelerated multimodal intervention in order to shorten the time of recovery after surgery has been reported [1-3]. The cost-effectiveness of clinical pathways including an accelerated perioperative care and rehabilitation intervention following total hip and knee arthroplasty (THA and TKA) has been shown [1].

Various reorienting acetabular osteotomies have been described [4-6]. The advantage of therapeutic exercise after periacetabular osteotomy (PAO) has been reported to promote the activity levels of patients to return to sports [7]. However, it has not been shown whether an accelerated perioperative care and rehabilitation intervention following PAO is effective.

We have performed PAO through an Ollier lateral U transtrochanteric approach since 1990 with consistent surgical indications and techniques [8]. We hypothesized that an accelerated protocol after PAO is effective for shortening the time of recovery and decreasing the rate of perioperative complications. The purpose of this retrospective study was to compare complication rates including the incidences of postoperative fractures in patients with standard and accelerated rehabilitation protocols who underwent PAO.

## Methods

The study design was approved by the Ethics Committee of Asahikawa Medical University. All investigations were conducted in conformity with ethical principles of research. Written informed consent for this study was obtained from all patients.

We retrospectively assessed all patients who had been managed using PAO between January 2002 and August 2011. During this period, 163 PAOs were performed in 145 patients for the treatment of acetabular dysplasia in adolescents and adults. All the patients reported moderate to severe hip pain. Surgical indications for the PAO included a lateral center-edge (CE) angle [9] of < 20° on anteroposterior radiographs, showing good joint congruency with the hip in abduction, pre- or early stage of grade 0, 1 or 2 osteoarthritis [10], and age younger than 60 years. Contraindications for the osteotomy were poor joint congruency showing partial narrowing or disappearance of the joint space with the hip in abduction, a severely deformed femoral head, advanced stage of Tönnis grade 3 osteoarthritis and age older than 60 years. In one patient (one hip), PAO was combined with an intertrochanteric valgus osteotomy. This patient was excluded from analysis because femoral osteotomies might affect the postoperative rehabilitation process. Six patients (six hips) were lost to follow-up. We evaluated the remaining 138 patients (156 hips). Nineteen patients were male and 119 were female. The left side was treated in 82 hips and the right side in 74 hips. Eighteen of the 138 patients underwent bilateral surgery. The average age of patients at the time of surgery was 30 years (range 11–59 years), and the average patient weight was 55.8 kg (range 39–83 kg). The operative techniques were described previously [8]. All of the procedures were performed by one surgeon who had performed more than 200 PAOs before the study periods. Complications were evaluated for 2 years.

During the study period, two rehabilitation protocols were applied (Table 1). For 76 consecutive hips in 65 patients between January 2004 and December 2008, an accelerated rehabilitation protocol was applied to achieve early postoperative recovery and functional improvement of patients. Postoperative strengthening of the hip, thigh and core musculature was begun on the day after surgery as tolerated. The exercise program included active hip range of motion, and gentle isometric hamstring and quadriceps muscle sets; these exercises were performed for 30 minutes in the morning and 30 minutes in the afternoon with a physical therapist every weekday for six weeks for all the patients. Full weight-bearing with two axillary crutches started on the day after surgery as tolerated. All weight-bearing exercises were performed under the guidance of physical therapists. Full weight-bearing was promoted using the weight scale by therapists. Muscle exercises of 1 hour with outpatient physical therapy continued 2 times per week for 3 months postoperatively.

**Table 1:** Comparison of standard and accelerated rehabilitation protocols

	**Standard protocol group (n = 80)**	**Accelerated protocol group (n = 76)**
Mobilization and exercise started	First postoperative day	The day of surgery
Partial weight-bearing with two crutches started	2–4 weeks	
Full weight-bearing with two crutches started	2 months	First postoperative day
Use of two crutches continued	Next 2 months	1 month
Use of one crutch continued	Next 3 months	Next 2 months

In the other period, a standard rehabilitation protocol was applied for 73 patients (80 hips). Since January 2009, a standard protocol has been applied again for all patients because postoperative pelvic fractures occurred frequently during the period in which the accelerated rehabilitation protocol was applied. The patient was allowed to use a wheelchair, and active range of motion, quadriceps and straight leg-raising exercises were begun on the first postoperative day. Non-weight-bearing walking with two crutches was also allowed as tolerated. Muscle exercises for 20 minutes per day with a physical therapist were performed every weekday for 6 weeks for all the patients. All weight-bearing exercises were performed under the guidance of physical therapists.

Prophylaxis against deep-vein thrombosis was not routinely administered for both groups. Only high-risk patients with a previous history of thrombosis were managed with aspirin for 2 weeks postoperatively.

Clinical and radiographic evaluations were performed preoperatively, and 1, 2, 4, 6, 12, 18 and 24 months postoperatively. The Harris hip score was also evaluated preoperatively and at the time of 2-year follow-up visits. Preoperative and postoperative clinical data were collected from charts of the patients.

Perioperative complications that occurred within 12 months after surgery included deep infection, pulmonary embolism, avascular necrosis of the acetabular fragment, displacement of the greater trochanter, fracture of the pubic ramus, ischial ramus or posterior column of the pelvis, nonunion of pubis and heterotopic ossifications. Fractures were defined as discontinuities of the bone other than the osteotomies that did not occur during surgery.

Conventional anteroposterior and lateral radiographs were obtained with the patient in the supine position for each evaluation. The CE angle, the acetabular head index (AHI) [11] and the acetabular angle of Sharp [12] were measured. The head lateralization index was measured [13]. The presence of the cross-over sign of acetabular retroversion was recorded. Gender, follow-up period, bilateral or unilateral involvement, severity of osteoarthritis, CE angle, angle of Sharp, AHI and head lateralization index were comparable between the standard and accelerated rehabilitation protocol groups (Table 2). Radiographic images were transferred to Image J software (National Institutes of Health, Bethesda, Maryland, USA) on personal computers, and measurements were performed with an accuracy of ±0.01 mm and ±0.01 degrees.

**Table 2:** Comparison of data on the patients, clinical outcomes, and radiographic evaluations in the standard and accelerated protocol groups

**Parameters**	**Standard protocol group (n = 80)**	**Accelerated protocol group (n = 76)**	**P value**
Age (range) (yr)	31.5 (11-59)	28.7 (14-58)	0.132
Sex (M: F) (no. of hips)	7:73	7:69	0.920
Side (left right) (no. of hips)	39:41	44:32	0.325
Harris hip score			
Preop.	68.5 ± 8.4	68.9 ± 8.5	0.220
Follow-up	91.6 ± 8.8	89.8 ± 9.3	0.133
Radiographic evaluation			
Center-edge (CE) angle (range) (°) [14]			
Preop.	-1.8 ± 9.5 (-28-19)	-1.7 ± 9.3 (-28-19)	0.977
Preop.	35.1 ± 6.1 (21-52)	35.6 ± 6.4 (22-50)	0.726
Acetabular head index (AHI) (range) [4]			
Preop.	54.1 ± 9.1 (23-72)	54.4 ± 8.6 (29-68)	0.917
Preop.	88.6 ± 7.3 (72-100)	88.5 ± 6.9 (74-100)	0.953
Sharp angle (range) (°) [15]			
Preop.	52.1 ± 3.4 (45-58)	51.5 ± 3.7 (47-56)	0.268
Preop.	40.2 ± 4.1 (30-48)	39.8 ± 3.9 (32-48)	0.431
Lateralization (range) (mm) [16]			
Preop.	16.0 ± 4.5 (9-30)	16.6 ± 3.9 (11-29)	0.288
Preop.	10.9 ± 6.7 (-4-24)	11.9 ± 6.3 (-2-22)	0.272
Perioperative complications	4	16	0.003

An orthopaedic instructor who specialized in hip surgery and imaging analyses of the hip joint made all radiographic measurements. Preoperative and periodic postoperative radiographic analyses were performed in a blinded fashion for all patients.

Univariate analyses included the chi-square test, the Mann–Whitney U test and the Wilcoxon signed rank test where appropriate. Preoperative and postoperative Harris hip scores were compared by the Wilcoxon signed rank test. The chi-square test was used for analyses of the clinical factors. The Wilcoxon signed rank test was used for analyses of preoperative and postoperative center-edge angle, acetabular head index, Sharp angle and head lateralization index. A probability value less than 0.05 was considered significant. Statistical analyses were performed using SPSS software, version 19.0 (SPSS Inc., Chicago, Illinois, USA).

## Results

The Harris hip score increased from a preoperative overall average of 69 points (range 52–95 points) to 91 points (range 54–100 points) at the most recent follow-up (P < 0.001). Overall, 146 hips (94%) had a hip score equal to or greater than 80 points. The mean Harris hip scores at preoperative and follow-up evaluation were similar in the two groups (Table 2). The overall mean CE angle increased (P < 0.001) from −2° ± 9° preoperatively to 35° ± 6° postoperatively, the mean AHI increased (P < 0.001) from 54 ± 9 to 89 ± 7 and the mean Sharp angle decreased (P < 0.001) from 52° ± 4° to 40° ± 4°. These improvements were similar in the two groups (Table 2). Preoperative cross-over signs were observed in 14 (9%) hips, which were absent postoperatively. The preoperative Tönnis grade of osteoarthritis was grade 0 in 39 (25%) hips, grade 1 in 114 (73%) hips and grade 2 in three (2%) hips. Three (2%) hips underwent THA.

The average period between the osteotomy and walking without support was 4.2 months (range 2.0-10.5 months) in 65 patients with the accelerated rehabilitation protocol and 6.9 months (range 2.5-15.0 months) in 73 patients with the standard rehabilitation protocol (P < 0.001), indicating that the accelerated rehabilitation protocol could achieve earlier recovery of patients.

**Figure 1:**
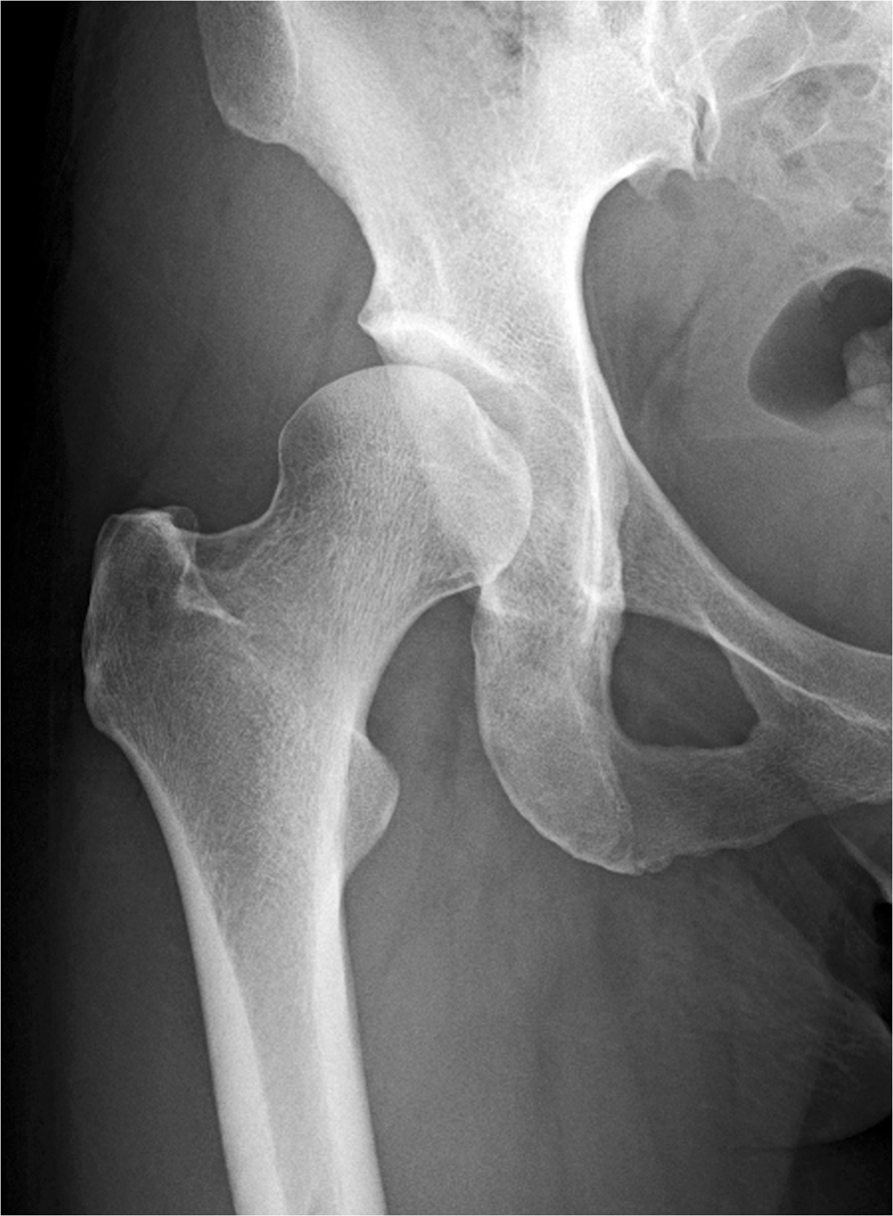
**Radiographic findings of a 39-year-old woman.** A preoperative radiograph showing acetabular dysplasia of the right hip.

**Figure 2:**
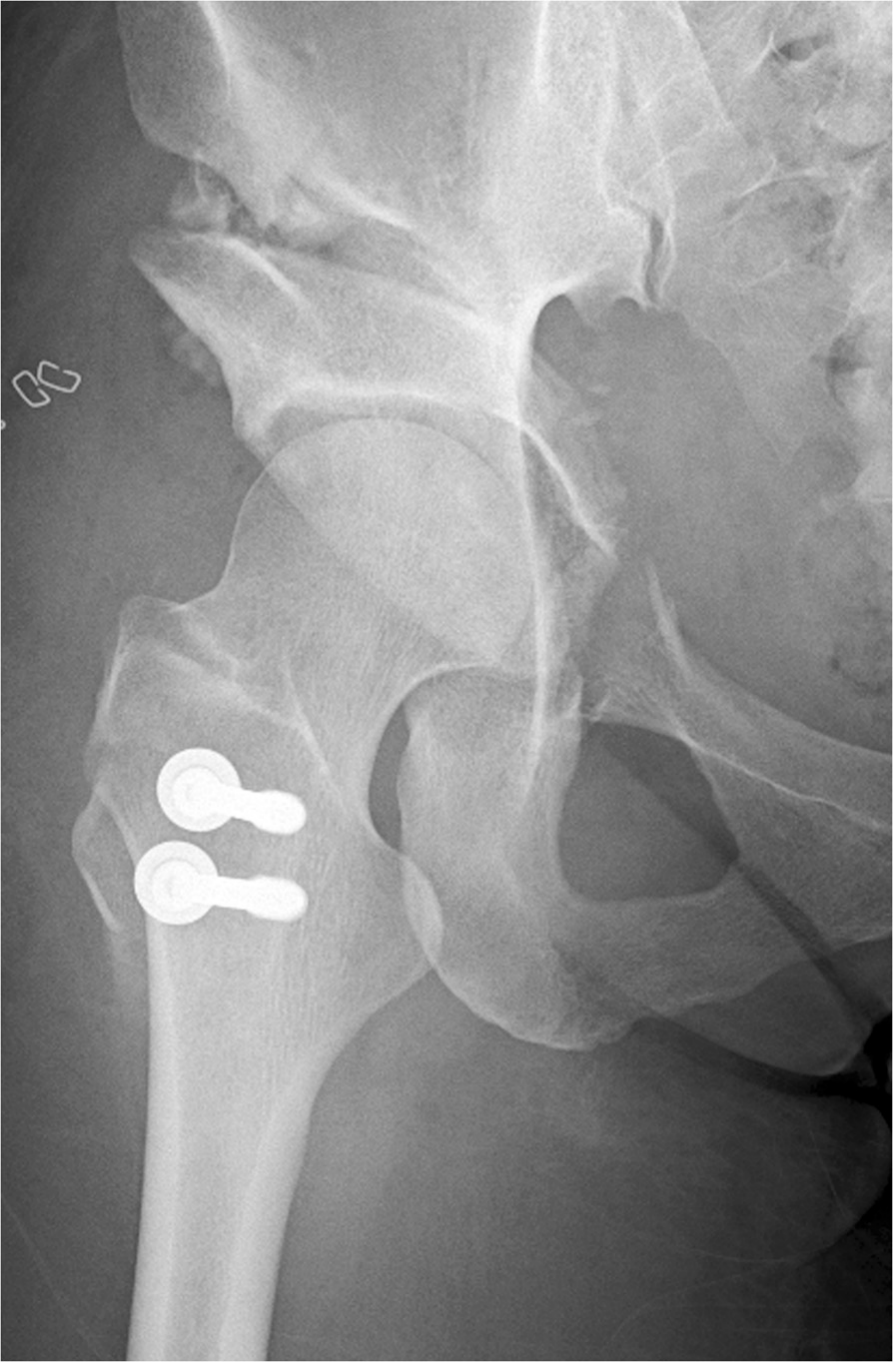
**A radiograph of the same patient made 1 week after periacetabular osteotomy showing good coverage of the femoral head without fracture of the posterior column.** The accelerated rehabilitation protocol was applied to the patient.

**Figure 3:**
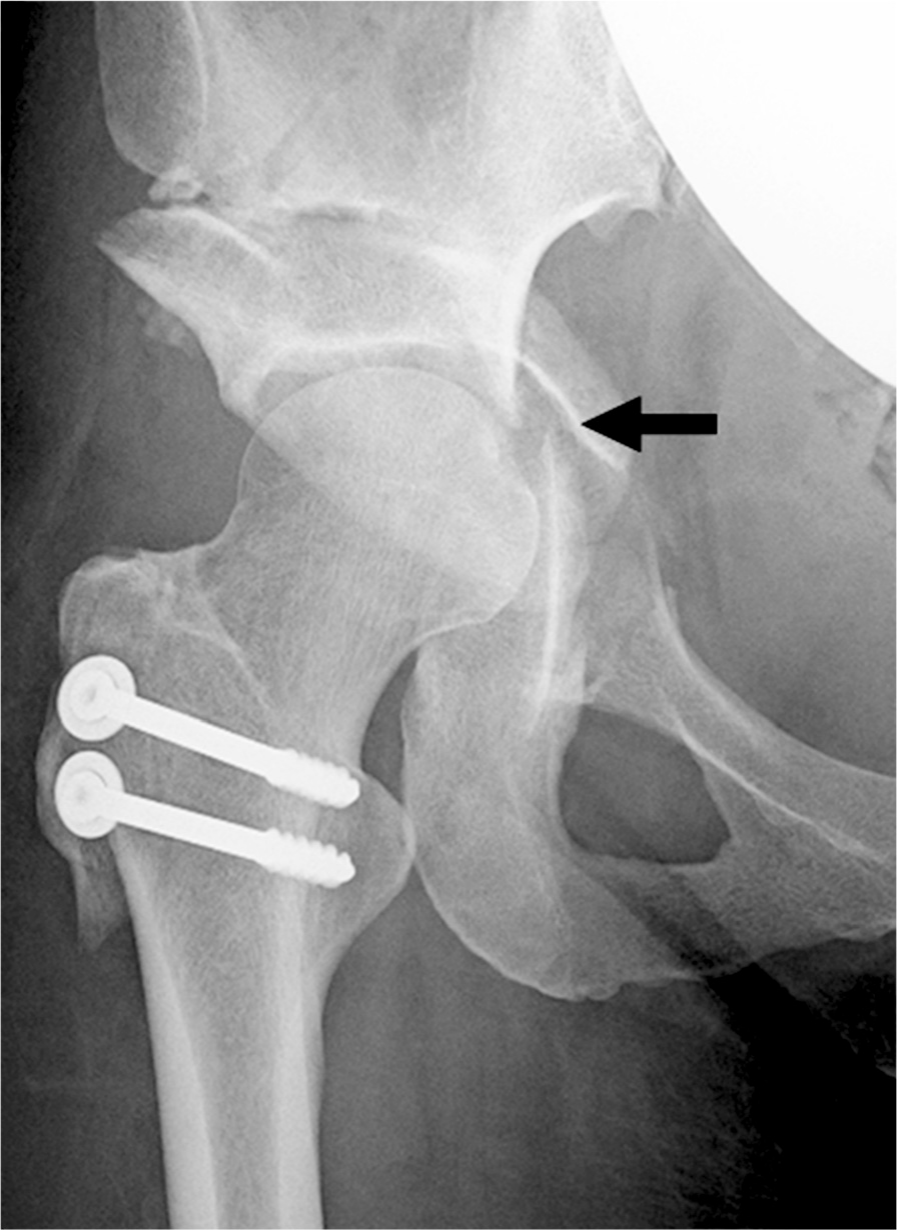
**A radiograph of the same patient made 4 weeks after osteotomy.** The patient reported right buttock pain 3.5 weeks postoperatively. A fracture of the posterior column (arrow) was revealed, which was not seen in Figure 1.

**Figure 4:**
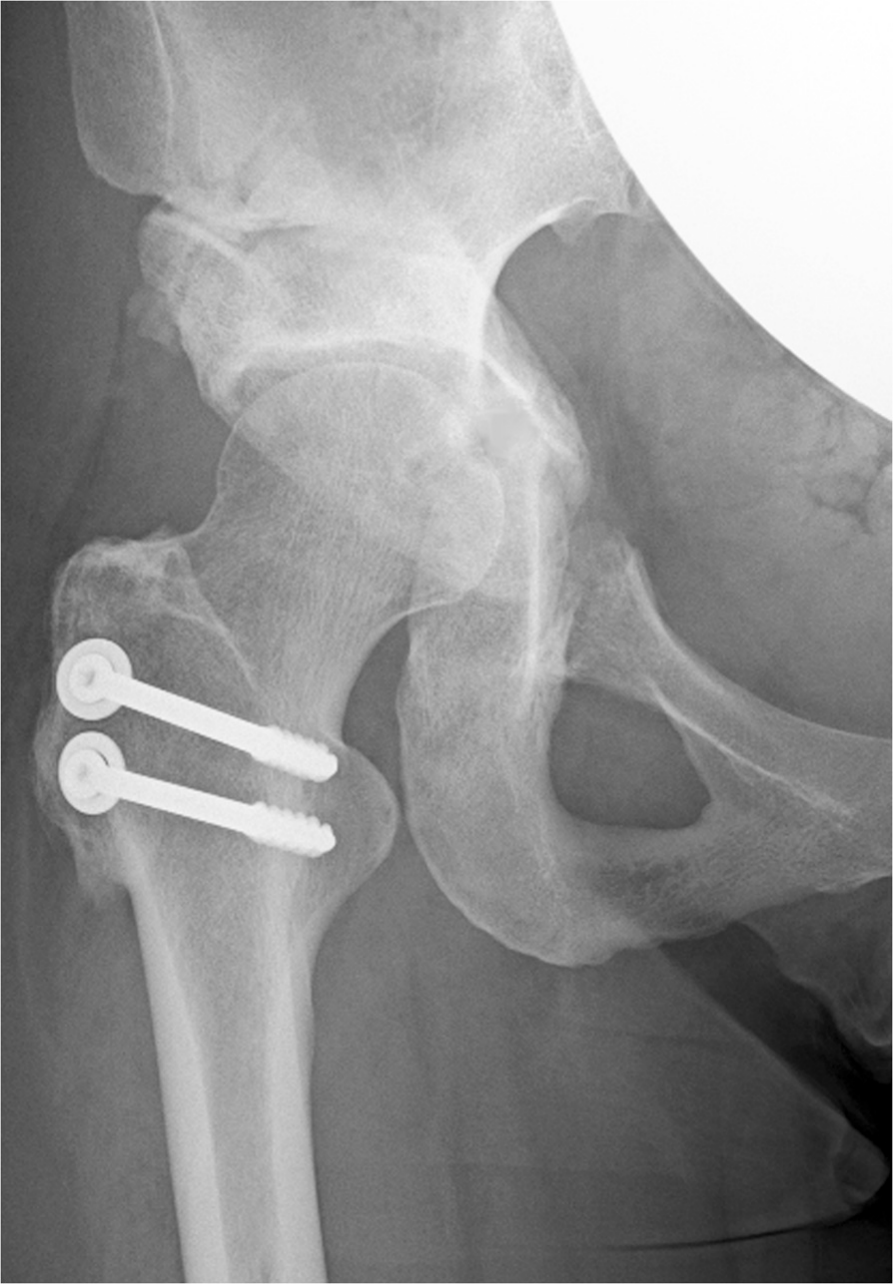
**A radiograph of the same patient made 2 years after osteotomy showing solid union of the fracture.** The right buttock pain continued for 3 months and then decreased. The patient reported no hip pain. The Harris hip score was 98 points at the time of follow-up.

There were no intraoperative fractures as determined by intraoperative inspection and postoperative radiographs in any patients. Postoperative fractures of the ischial ramus and posterior column of the pelvis were more frequently found in patients with the accelerated rehabilitation protocol (8/76) than in those with the standard rehabilitation protocol (1/80) (P = 0.013). Other perioperative complications were similar in the two groups (Table 3). Postoperative fractures occurred at an average of 2.0 months (range 0.2-12 months) postoperatively. Slight pain or dullness continued for an average of 3.5 months (range 2.0-12.0 months) in patients with fractures. The mean time to union of ischial fracture was 9.0 months. All fractures united without surgical intervention (Figures 1, 2, 3, 4). There was no loss of corrections. One postoperative deep infection extended to septic arthritis, which healed by surgical debridement. One hip with displacement of the greater trochanter was revised and refixed using a metal cable grip system. Bone union could be obtained. No patient had an injury to the great vessels or major nerves. No patient had symptoms resulting from damage to the lateral femoral cutaneous nerves.

**Table 3:** Comparison of perioperative complications in the standard and accelerated protocol groups

	**Standard protocol group (n = 80)**	**Accelerated protocol group (n = 76)**	**P value**
Deep infection	1	0	0.513
Osteonecrosis of the acetabular fragment	1	2	0.530
Displacement of the greater troachanter	0	1	0.303
Fracture of the public ramus	0	1	0.303
Fracture of the ischial ramus	0	3	0.073
Fracture of the posterior column of the pelvis	1	5	0.072
Asymptomatic public non-union	1	4	0.155
Asymptomatic heterotopic bone formation	0	1	0.303

## Discussion

Many authors have reported success with accelerated rehabilitation protocols after THA and TKA [1-3]. Immediate mobilization on the day of surgery has been reported to decrease the length of stay without adverse effects on complications or readmissions. A previous randomized clinical trial showed that an accelerated perioperative care and rehabilitation protocol can be both cost-saving and effective, with a reduction in the length of hospital stay and a gain in health-related quality of life [2]. However, it has not been clear whether accelerated rehabilitation for patients undergoing PAO is also effective.

The average period between the osteotomy and full weight-bearing walking without support was shorter in patients with the accelerated rehabilitation protocol in this study. This indicates that the accelerated rehabilitation protocol had advantages for early muscle recovery in patients undergoing PAO.

However, the incidence of postoperative pelvic fractures was higher in patients with the accelerated rehabilitation protocol. Patients with PAO may exhibit several different features compared with those with THA. Because PAO is osteotomy surgery, proper time is necessary for pelvic bone union and healing. There is no need for bone union or healing in most total joint replacements. It seems that this is the fundamental difference between these two surgical procedures. The load transmission patterns through the pelvic ring change soon after PAO. Kaku et al. reported that load transfer through the pelvis is higher in the superior pubic ramus than in the inferior ramus [17]. Because the load can be transferred only through the inferior pubic ramus and the ischium after PAO, increased load might cause high strain, which results in stress fractures of the ischial ramus postoperatively. The load transmitted through the posterior column of the pelvis also increased soon after PAO. These increases in load seemed higher in patients with the accelerated rehabilitation protocol, especially for several weeks postoperatively, which might have caused high fracture rates of the ischial ramus and the posterior column.

Espinosa et al. described that the incidence of ischial fractures was extremely low (0.9%) and suggested that the polygonal shape of the PAO is not the root cause of ischial fractures [18]. They discussed that substantial weakening of the bone could occur while performing the ischial cuts during the PAO. Fractures of the ischial ramus and the posterior column of the pelvis occurred in three of 210 hips with the standard rehabilitation protocol in the present study, which coincides with their results. In contrast, those fractures occurred in seven of seventy-six hips with the accelerated rehabilitation protocol, which was much higher than the rate with the standard rehabilitation protocol. This fracture rate was unacceptably high compared with those in previous studies (Table 4). All fractures in this study healed uneventfully with nonoperative treatment. Because there were no documented injuries preceding the fractures, it must be assumed that normal loads were sufficient to cause stress fractures in patients with the accelerated rehabilitation protocol. Although postoperative fractures of the ischial ramus and posterior column of the pelvis were more frequently found in patients with the accelerated rehabilitation protocol, they do not seem to have influenced the two-year outcomes after PAO.

**Table 4:** Literature review of postoperative fixation loss or fracture after periacetabular osteotomies

**Author**	**Number of hips**	**Male: female (no. of hips or patients)**	**Partial weight-bearing**	**Full weight-bearing**	**Fixation loss, fractures of the pubis, ischium or posterior column (no. of hips)**
Trousdale et al. [19] (1995)	42	8:34	3 days	2 months	0
Siebenrock et al. [15] (1999)	75	17:58	3 days	2 months	2
Matta et. [20] (1999)	66	42:16	1 day		1
Crockarell et al. [21] (1999)	21	14:5	3 days	8-10 weeks	3
Ko et al. [22] (2002)	38	1:37	1 week	3-4 months	0
Espinosa et al. [18] (2002)	526		6-8 weeks		17
Nozawa et al. [23] (2002)	50	46:3	2 months	4-6 months	1
Hsieh et al. [24] (2003)	46	30:8	4-5 days	3 months	0
Yasunaga et al. [13] (2003)	89	8.3	6 weeks	5-6 months	0
Matheney et al. [16] (2009)	135	119:16	1 day	6-10 weeks	0
Yamaguchi et al. [25] (2010)	164		1 day	4 months	0
Teratani et al. [14] (2010)	96	2:94	3 days	2 months	0
Current study (patients with accelerated protocol)	76	7:69	1 day	1 day	9

Our study has several limitations. Our study design was retrospective and included a relatively small number of patients, which limits the statistical power. Our results are representative only of Asian patients with short stature and low body mass index. They may not be applicable to Caucasian patients.

## Conclusion

The accelerated rehabilitation protocol seems to have advantages for early muscle recovery in patients undergoing PAO; however, postoperative pelvic fracture rates were unacceptably high in patients with this protocol. It is still unclear whether it is worth applying the accelerated rehabilitation protocol after PAO. These fractures do not seem to influence long-term clinical results after PAO. Surgeons must be aware that postoperative pelvic fracture rates would increase if they apply the accelerated rehabilitation protocol. We now prefer the standard rehabilitation protocol for patients undergoing PAO.

## Abbreviations

PAO: Periacetabular osteotomy; THA: Total hip arthroplasty; TKA: Total knee arthroplasty; CE angle: Center-edge angle; AHI: Acetabular head index.

## Competing interests

The authors declare that they have no competing interests.

## Authors’ contributions

HI assembled and analyzed the data, and wrote the manuscript. HT, TS and YN collected clinical follow-up data and analyzed the data. HI and TM performed the surgery, recorded the intraoperative data, and arranged intraoperative photography. TM was the head of department and principal investigator. All authors red and approved the final manuscript.

## Pre-publication history

The pre-publication history for this paper can be accessed here:

http://www.biomedcentral.com/1471-2474/15/234/prepub
